# Endocrine markers of energy deficiency and within-day energy balance metrics in elite athletes

**DOI:** 10.3389/fspor.2026.1894602

**Published:** 2026-08-12

**Authors:** J. Trinh, P. H. Nilsson, H. Pojskic, M. Ahlgren, C. Mohlin, P. Pagels, T. Ragnarsson, D. Samyn, K. Tryselius, A. K. Melin

**Affiliations:** 1 Department of Molecular and Clinical Medicine, Institute of Medicine, University of Gothenburg, Gothenburg, Sweden; 2 Department of Chemistry and Biomedicine, Linnaeus University, Växjö/Kalmar, Sweden; 3 Department of Sport Science, Linnaeus University, Växjö/Kalmar, Sweden; 4 School of Medicine, Faculty of Medicine and Health, Örebro University, Örebro, Sweden; 5 Department of Laboratory Medicine, Clinical Chemistry, Örebro University Hospital, Örebro, Sweden; 6 Department of Health and Caring Sciences, Linnaeus University, Växjö/Kalmar, Sweden

**Keywords:** dietary characteristics, energy availability, hormones, metabolic markers, REDs

## Abstract

**Introduction:**

This cross-sectional study aimed to determine the prevalence of Relative Energy Deficiency in Sport (REDs) among Swedish national-team athletes across multiple sports and to examine selected endocrine and metabolic markers in relation to within-day energy balance (WDEB) metrics.

**Methods:**

REDs severity was retrospectively classified in 43 athletes (60.5% females) using the IOC REDs Clinical Assessment Tool 2 framework. Assessments included DXA-derived body composition, resting metabolic rate (RMR) by ventilated-hood indirect calorimetry, blood markers, validated questionnaires, clinical eating disorder interview. 4-day dietary intake, energy availability (EA), EB, and WDEB metrics were assessed in a subsample (*n* = 33; 19 females, 14 males).

**Results:**

Twenty athletes (46.5%) were classified as controls (no/low risk; 45.0% female), whereas 23 (53.5%) were classified with REDs (73.9% female). Most REDs cases were mild, however 61% exhibited ≥2 additional potential REDs indicators, a pattern also observed in 50% of controls. Athletes classified with REDs had higher cortisol and cortisol-to-insulin ratio, lower RMR ratio, lower energy intake and 24 h EA, and greater exposure to negative WDEB patterns. In unadjusted analyses, insulin was associated with both 24 h EB and EA. Exploratory analyses in females identified associations between cortisol-to-insulin ratio and negative WDEB metrics, and between Exercise Addiction Inventory score and 24 h EB; however, these associations were no longer significant after Benjamini-Hochberg false discovery rate correction.

**Conclusion:**

More than half of the elite athletes in this cohort, were classified with REDs, with a higher prevalence among females. REDs classification was associated with lower EA, greater exposure to negative WDEB patterns, and additional endocrine and metabolic alterations consistent with energy deficiency. Notably, a similar pattern was also observed in 50% of controls, supporting a continuum rather than discrete risk categories. Although WDEB and dietary metrics may provide useful contextual information they should be interpreted cautiously and integrated with comprehensive multi-marker clinical REDs evaluation.

## Introduction

Elite athletes are exposed to high, and often fluctuating training and competition demands, creating substantial energy and macronutrient requirements that may be challenging to meet consistently. Under-fueling can occur intentionally through restrictive eating, weight-management practices, or disordered eating behaviors, or unintentional, when dietary intake fails to adequately compensate for exercise energy expenditure (EEE) and recovery requirements (1, 2). In addition, appetite regulation may be disrupted, leading to insufficient compensatory increases in energy intake during periods of intensified training (3, 4). Habitual dietary practices may further contribute to in intake, particularly when diets are characterized by low energy density due to high fiber intake or limited consumption of fat and carbohydrate-rich foods (5, 6). Collectively, these factors can result in sustained low energy availability, whereby insufficient energy remains to support the physiological functions necessary for health, recovery and athletic performance (2, 7).

Energy availability (EA), defined as dietary energy intake minus EEE and normalized to fat-free mass (FFM) (8)*,* reflects the energy remaining to support essential physiological functions including cellular maintenance, thermoregulation, growth, reproduction, and immune function (2, 8). As energy resources are limited, greater expenditure on one process such as exercise, reduces the energy available for others physiological functions, such as reproduction (9). While short-term or moderate exposure to low EA (LEA) may have limited or reversible consequences, prolonged or severe LEA can result in Relative Energy Deficiency in Sport (REDs), a syndrome characterized by widespread disruption in metabolic, endocrine, hematological and other physiological systems (2). REDs has been linked to an elevated risk of injury (10–13), and impaired sport performance (14–17).

A recent meta-analysis of 46 studies involving 6 118 athletes reported a mean prevalence of 45% for LEA and REDs (18), highlighting the widespread nature of this nutrition-related syndrome in athletic populations. However, important gaps remain in our understanding of REDs particularly regarding standardization, validity and practical application of EA assessment methods (2, 7, 19, 20).

EA is distinct from energy balance (EB), which reflects the relationship between energy intake and total energy expenditure. Within-day EB (WDEB) extends this concept by characterizing the timing, magnitude and duration of energy deficits and surpluses across the day (21, 22). While 24 h EA and EB calculations provide useful theoretical and research frameworks, their assessment in free-living athletes is constrained by substantial methodological and biological challenges, including the accurate measurement of dietary intake, EEE, and the cumulative effects of energy deficiency relevant to current health and performance outcomes (7, 19, 23).

Endocrine and metabolic biomarkers can provide important biological insight into the continuum of energy deficiency; however, their interpretation in athletes remain complex and biomarker specific. Experimental and field based studies demonstrate that energy scarcity may affect reproductive, thyroid, metabolic, and stress-related pathways, although the responses vary by biomarker assessed, study design, participant characteristics, sex, training load, dietary composition, and duration of exposure (2, 7, 24, 25). In addition, field studies frequently fail to identify consistent thresholds or clear associations between self-reported EA and objective indicators of energy conservation or reproductive disturbance (7, 26, 27). Therefore, endocrine and metabolic biomarkers should be interpreted as part of an integrated multi-marker clinical assessment rather than independent diagnostic indicators of REDs (2, 23, 28).

Furthermore, interpretation of many endocrine and metabolic biomarkers should be sex specific. In female athletes, menstrual and ovulatory function, estradiol concentration and hormonal contraceptive are critical considerations when evaluation reproductive and endocrine function. Consistent with both the REDs and Female Athlete Triad frameworks, disruption of hypothalamic-pituitary-ovarian axis signaling remains a clinically important consequence of persistent energy deficiency (2, 20, 28). In male athletes, assessment of testosterone, libido, morning erections, bone health and other indicators of hypothalamic-pituitary-gonadal axis function can provide valuable clinical insight. Nevertheless, evidence-based EA thresholds and biomarker cutoffs for males remain less established than those established in female-focused research (2, 28). Available data in male athletes suggest heterogeneous responses, with short-term or episodic exposure often providing limited measurable effects, while more sustained or severe exposure may result in testosterone suppression, reductions in IGF-1 and insulin and adverse psychological and behavioral outcomes, including reduced well-being, cognitive restraint, heightened cognitive restraint, and depressive symptoms (29–32).

A limitation of 24 h EA and EB models is that daily averages may mask prolonged or repeated periods of within-day deficits. To address this limitation, WDEB analysis estimates hourly EB and characterizing the timing, magnitude and duration of energy deficits and surpluses (21, 22). In female endurance athletes, functional hypothalamic amenorrhea has been associated with greater time spent in negative WDEB despite similar 24 h EA and EB, and with within-day deficits associated with lower resting metabolic rate (RMR), lower estradiol concentrations and higher cortisol levels (33). Comparable associations have been reported in male endurance athletes, where greater exposure to severe within-day energy deficit, has been associated with suppressed RMR, higher cortisol and a lower testosterone-to-cortisol ratio (34). Additional evidence from elite swimmers, links adverse WDEB patterns and late-day energy intake with lower total T3 concentrations, and metabolic compensation, whereas findings in female soccer players suggests that common LEA-risk indicators may not adequately reflect measured EA or WDEB (35, 36). Collectively, these findings suggest that WDEB may provide a complementary contextual approach that may enhance the interpretation of 24 h EA, EB, clinical assessment and endocrine outcomes.

Despite advances in REDs research, clinical risk stratification and biomarker methodology, limited evidence has integrated WDEB metrics, endocrine and metabolic biomarkers, and the International Olympic Committee (IOC) REDs Clinical Assessment Tool (CAT) 2-informed risk/severity classification in elite female and male athletes from multiple sports (28, 37). Addressing this gap may improve understanding of the relationship between the within-day energy status, endocrine adaptations, and clinical manifestation of REDs while informing sex-specific monitoring and management strategies in high performance sport.

Therefore, the aims of this cross-sectional study were to determine the prevalence of REDs among Swedish national-team athletes from multiple sports and to evaluate selected endocrine and metabolic biomarkers in relation to WDEB metrics.

## Materials and methods

This cross-sectional observational two-step study was part of the REDs Sweden study (ClinicalTrials.gov ID: NCT06371963) conducted in accordance with the Helsinki Declaration and approved by the Swedish Ethical Review Authority (No: 2021-07031-01).

### Participants

**Figure 1:**
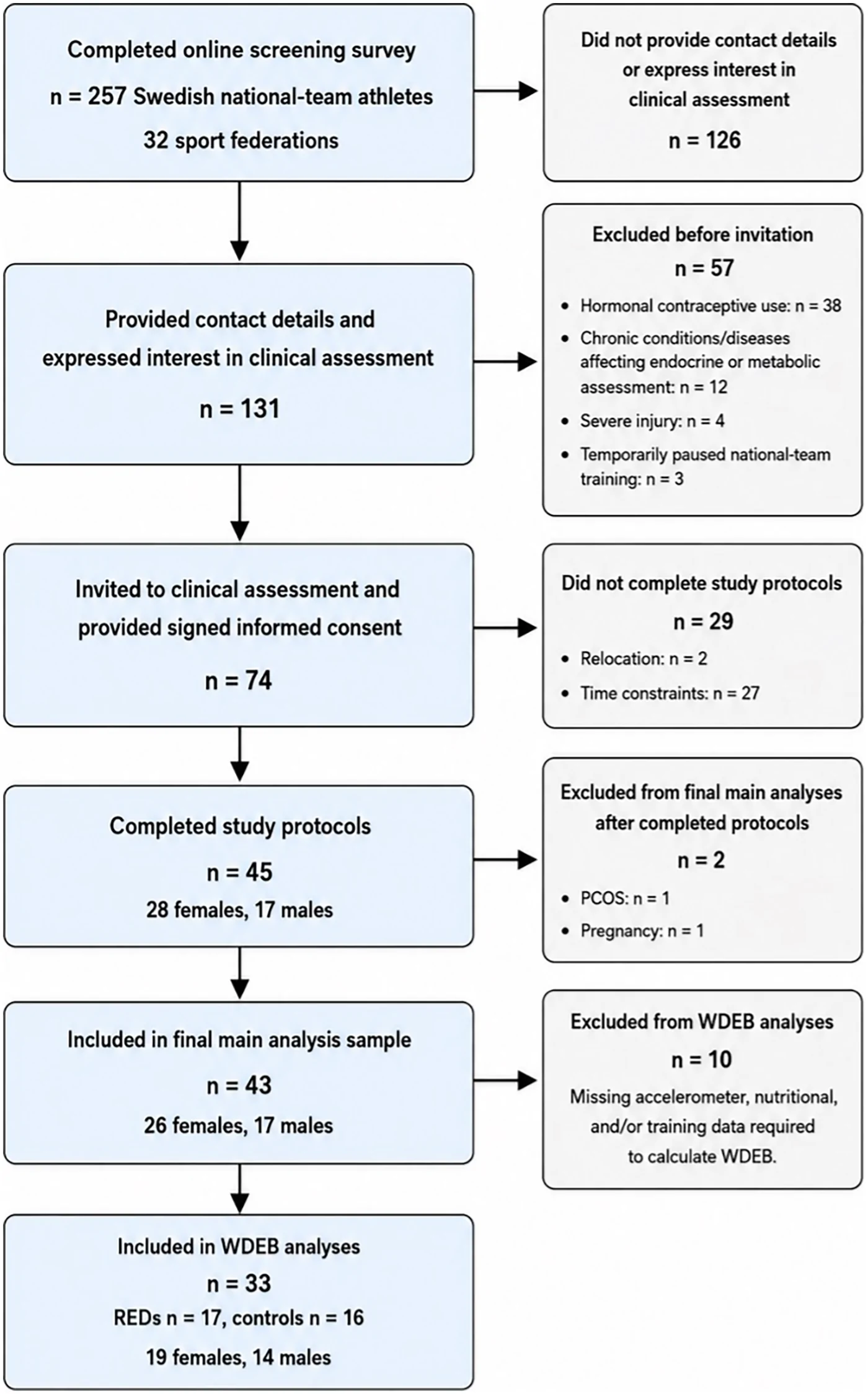
Recruitment flowchart of Swedish national-team athletes. Of 257 athletes from 32 sport federations who completed the online screening survey, 131 provided contact details and expressed interest in clinical assessment, while 126 did not progress. After pre-invitation exclusions (*n* = 57), 74 athletes provided informed consent; 29 withdrew, leaving 45 who completed study procedures. Two were subsequently excluded due to PCOS (*n* = 1) and pregnancy (*n* = 1), resulting in a final main analysis sample of 43 athletes (26 female, and 17 male athletes). Ten athletes were excluded from WDEB analyses due to missing accelerometer, nutritional, and/or training data, leaving 33 athletes for WDEB analyses (REDs *n* = 17, controls *n* = 16; 19 female and 14 male athletes). WDEB, within-day energy balance; REDs, Relative Energy Deficiency in Sport; PCOS, polycystic ovary syndrome.

Participants were recruited through the Swedish Olympic Committee, the Swedish Sport Federations, and targeted social media campaigns to the first phase of the study, which consisted of an anonymous online survey assessing self-reported symptoms of REDs and disordered eating behaviors. In total, 257 Swedish national-team athletes (tier 4 (38) from 32 sport federations completed the survey (Figure 1). Among these athletes, 131 (71.2% female) provided contact information and expressed interest in participating in the second phase, which included clinical assessment of endocrine and metabolic status, bone health and eating behaviors, followed by assessment of dietary intake and energy expenditure. Of the eligible 131 athletes, 57 were excluded: 38 female athletes because of hormonal contraceptive use, 12 athletes because of chronic conditions known to influence endocrine or metabolic outcomes [e.g., polycystic ovary syndrome (PCOS), primary hypothyroidism, and inflammatory bowel disease], four because of severe injury, and three because of a temporarily cessation of national team training. Consequently, 74 athletes were invited to participate in the clinical assessments. Prior to participation, all athletes received verbal and written study information and provided written informed consent. The targeted sample-size was based on detecting clinically relevant differences in hormones concentrations (estradiol/testosterone) with 80% power and was informed by previous research (11). The power calculation indicated that at least 14 female athletes and 8 male athletes were required per comparison group.

**Figure 2:**
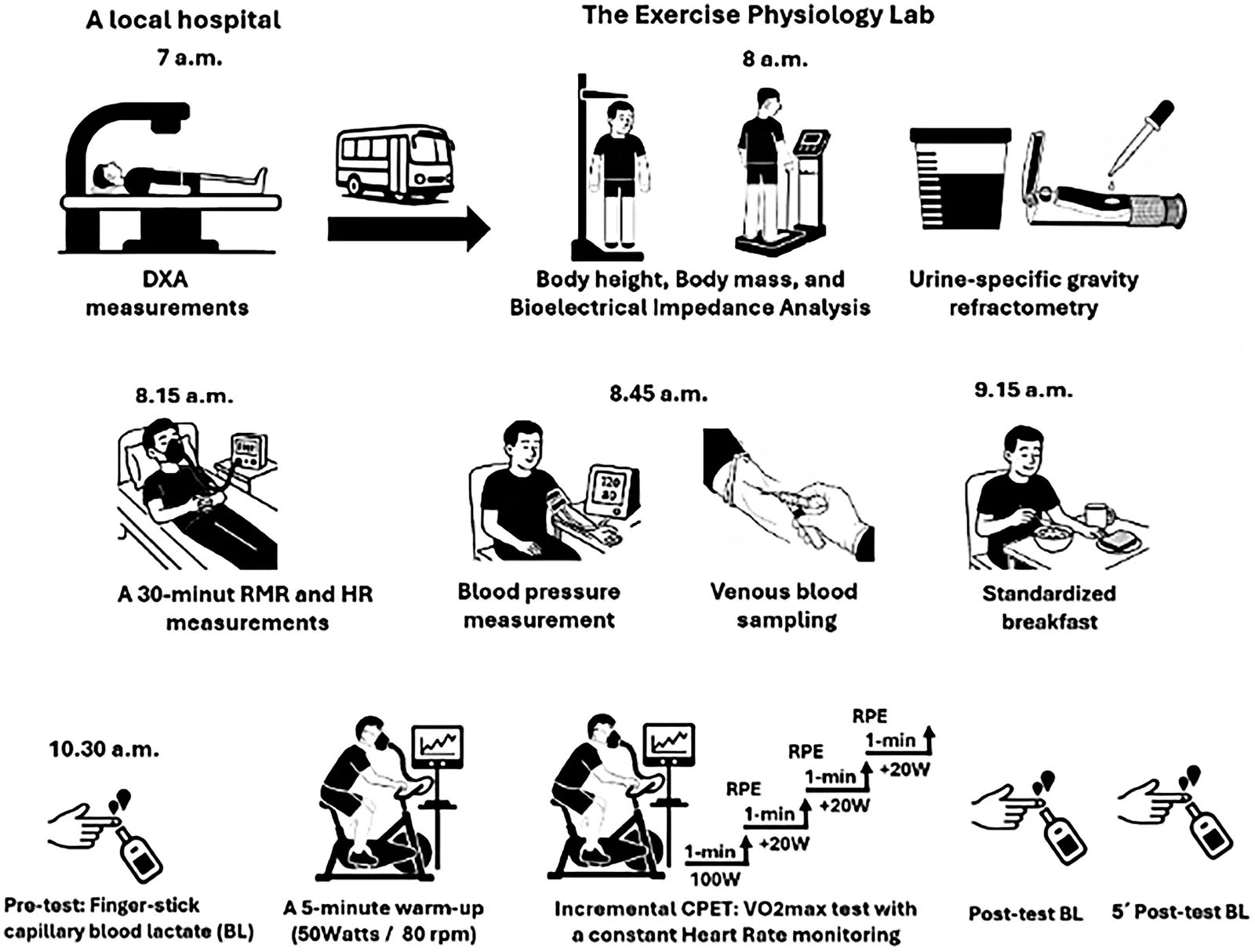
Experimental protocol flowchart. BL (blood lactate); DXA (dual-energy x-ray absorptiometry); RMR (resting metabolic rate). Adapted from Pojskic et al. (61), licensed under CC BY 4.0.

Of the 131 invited athletes, 74 withdrew. Two because of relocation and 27 because of time constraints. As a results, 45 national-team athletes completed the study protocols (28 female and 17 male) from various sport disciplines completed the study protocols (see Table 3). Two participants were subsequently excluded from all analyses because of PCOS (*n* = 1) and pregnancy (*n* = 1), yielding a final analytic sample of 43 athletes (26 females and 17 males; Figure 2).

Assessments were individually scheduled during the athletes' preparatory training period. Menstruating participants were tested in the early follicular phase (days 2–5 of the menstrual cycle) (39), whereas participants with amenorrhea were assessed when independent of cycle phase. Because recent exercise can influence endocrine and metabolic biomarkers, including cortisol, testosterone, insulin and IGF-1, participants were instructed to refrain from training on the day before testing (40–43). This procedure provided a minimum washout period of 36 h between the last training session and blood sampling, which is generally considered sufficient to minimize acute exercise on these markers (44–48). Blood samples were collected in the early morning after an overnight fast of at least 12 h. Participants arrived at the laboratory in a rested state, and those residing outside the region stayed overnight near the testing facilities and standardize testing conditions.

#### Menstrual status and contraceptive use

Female athletes using hormonal contraceptives were excluded before invitation to the clinical assessment, as described above, to minimize confounding in the classification of menstrual-function classification and the interpretation of reproductive-hormone concentration. During a three-month monitoring period, naturally menstruating participants recorded the first day of each menstrual bleeding episode and tracked menstrual cycle length using a commercially available period-tracking application of their choice. In addition, participants perform daily urinary luteinizing hormone (LH) testing using ovulation test strips (RFSU AB, Kista, Sweden), starting on day seven of the menstrual cycle and continuing until a positive result was obtained.

Eumenorrhea was defined as a menstrual cycle length between 21 and 35 days, in the absence of pregnancy, polycystic ovary syndrome (PCOS), hyperprolactinemia, or another clinically confirmed cause of menstrual dysfunction. Oligomenorrhea was defined as a menstrual cycle length >35 days. Secondary amenorrhea was defined as the absence of menstrual bleeding for ≥3 consecutive cycles, whereas prolonged secondary amenorrhea was defined as the absence of menstrual bleeding for ≥12 consecutive cycles, in the absence of pregnancy, PCOS, or hyperprolactinemia. Ovulatory status was assessed separately from menstrual-cycle length. Anovulatory cycles were defined as the absence of a positive LH test result in at least two of the three monitored cycles (39) and was considered a potential indicator of REDs, and was classified accordingly in the risk assessment framework (Table 1).

**Table 1:** Primary and secondary indicators and risk stratification of REDs used in the present study.

Indicator	Group	Methods and operational definition/threshold
Severe primary (scored as 2 indicators)		
Primary amenorrhea	Females	LEAF-Q: no menarche
Prolonged secondary amenorrhea	Females	LEAF-Q, interview and blood analysis: no menstrual bleeding ≥12 consecutive months in absence of pregnancy, PCOS, and prolactin >620 mIE/L
Clinical low free or total testosterone	Males	Blood analysis: <4.1 nmol/L for males 16–21 yrs and <5.7 nmol/L for males >21 yrs of age
Primary (scored as 1 indicator)		
Secondary amenorrhea	Females	LEAF-Q, interview and blood analysis: no menstrual bleeding for 3–11 consecutive months in absence of pregnancy, PCOS, and prolactin >620 mIE/L
Eating disorders	Females	EDE-Q global score ≥2.30 and EDE interview
	Males	EDE-Q global score ≥ 1.68 and EDE interview
Subclinical low free or total testosterone	Males	Blood analysis: <11.3 nmol/L for males 16–21 yrs and <10.3 nmol/L for males >21 yrs
Low thyroid hormones	All	Free T3 < 4.3 pmol/L and/or free T4 < 13.0 pmol/L with TSH <4,8 mIE/L
Low BMD	All	DXA Z-score ≤−1 at the lumbar spine, total hip, or femoral neck
Secondary (scored as 1 indicator)		
Oligomenorrhoea	Females	LEAF-Q, interview and blood analysis: >35 days between menstrual cycles; max 8 menstrual cycles/yr with absence of pregnancy, PCOS, and prolactin >620 mIE/L
Bone stress injury history	All	On-line survey: history of ≥1 BSI within the previous 2 yrs
Elevated total or LDL cholesterol	All	Blood analysis: total cholesterols > 6.1 mmol/L for those ≤30 yrs and > 6,9 mmol/L for those >30 yrs
Depression symptoms	All	Major Depression Index total score ≥26
Potential (not scored)		
Anovulation	Females	≥2 anovulatory menstrual cycles within a 3-month period of urinary ovulation detection (luteinizing hormone test strips)
GI symptoms	Females	LEAF-Q GI score ≥2
Low libido	Males	LEAM-Q libido score ≥2
Low IGF-1	All	Blood analysis: <21 yrs <128.5 μg/L; 22–24 yrs <146.5 μg/L; 25–29 yrs 127.8 μg/L; 30–34 yrs 111.8 μg/L; 35–39 yrs 103.0 μg/L; 40–44 yrs 98.3 μg/L
Low insulin	All	Blood analysis: <3 mIE/L
High cortisol	All	Blood analysis: >800 nmol/L
Low glucose	All	Blood analysis: <4 mmol/L
Low ferritin	All	Blood analysis: females <10 µg/L, males <20 µg/L
Low RMR	All	RMR ratio <0.90 or RMR <30 kcal/kg FFM/day
Bradycardia	All	Heart rate <40 beats/min
Low blood pressure	All	Blood pressure <90/60 mm Hg
Exercise dependence	All	Exercise Addiction Inventory >24
Low BMI	All	BMI <18.5 kg/m^2^

BMD, bone mineral density; BMI, body mass index; BP, blood pressure; BSI, bone stress injury; DXA, dual-energy x-ray absorptiometry; EDE-Q, Eating Disorder Examination Questionnaire; EDE interview, Eating Disorder Examination Interview; GI, gastrointestinal; IGF-1, insulin-like growth factor-1; HR, heart rate; LDL, low-density lipoprotein; LEAF-Q, Low Energy Availability in Females Questionnaire; LEAM-Q, Low Energy Availability in Males Questionnaire; PCOS, poly cystic ovary syndrome; RMR, resting metabolic rate; T3, triiodothyronine; T4, thyroxine.

### Clinical procedures

The initial online survey included demographic questions and validated psychometric instruments, including the Eating Disorder Examination Questionnaire 6.0 (EDE-Q; 49), the Major Depression Index (MDI; 50), and the Exercise Addiction Inventory (EAI; 51). To facilitate REDs screening, male athletes completed libido related items derived from the Low Energy Availability in Males Questionnaire (LEAM-Q) for which a score of ≥2 was considered indicative of risk of REDs (32). Female athletes completed menstrual and gastrointestinal function items derived from the Low Energy Availability in Females Questionnaire (LEAF-Q) (52), with a total score ≥8 used to identify athletes at risk of REDs.

#### Eating disorder examination questionnaire (EDE-Q 6.0)

The EDE-Q (49) measures the core pathology of eating disorders over the past 28 days and can assess symptoms of eating disorders in both male and female athletes (53). It consists of 28 items distributed across four subscales: Restraint, Eating Concern, Shape Concern and Weight Concern. Responses are recording using a 7-point Likert scale ranging from: (1) No days, (2) 1–5 days, (3) 6–12 days, (4) 13–15 days, (5) 16–22 days, (6) 23–27 days and (7) every day (54). Each subscale score is calculated by summing item ratings and dividing by the number of items in that subscale. The EDE-Q global score is derived by averaging the four subscale scores. Consistent with established sex-specific cutoffs, a global score ≥1.68 in males (55) and ≥2.30 in females (56) was considered indicative of elevated eating-disorder symptomatology. Higher scores reflect a greater degree of eating disorder pathology (54, 57). In the present study, these sex-specific EDE-Q global score thresholds were used in the initial screening to identify athletes at increased risk of REDs, and to indicate clinically relevant symptoms of eating disorders.

#### Major depression inventory (MDI)

The MDI is a questionnaire designed to assess depressive symptomatology and is aligned with the diagnostic criteria for depression in the Diagnostic and Statistical Manual for Mental Disorders, IV-Text Revision (DSM-IV-TR; 50). The instrument consists of 10 items that evaluate depressive symptoms experienced during the preceding 2 weeks. Each item is rated on a six-point Likert scale ranging from 0 (never) to 5 (all the time) with higher scores indicating greater symptom severity. Responses are summed to generate a total score ranging from 0 to 50. Consistent with established recommendations, a total score ≥26 was used in the present study to identify participants reporting clinically relevant symptoms of depression (50).

#### Exercise addiction inventory (EAI)

The EAI is a brief questionnaire consisting of six items developed to identify individuals at risk of exercise addiction (51). Each of the six items reflects core components of addictive behavior and is rated on a five-point Likert scale ranging from 1 (*strongly disagree*) to 5 (*strongly agree*). Item scores are summed to produce a total score ranging from 6 to 30, with higher scores indicating greater risk of exercise addiction. Consistent with established recommendations, a total score of ≥24 was used in the present study to identify athletes being at risk for exercise addiction (58).

#### Eating disorder examination interview

The Eating Disorder Examination (EDE) Interview (59) is a semi-structured, clinician-administered interview used to assess DSM-5 eating disorder psychopathology and establish operational eating disorder diagnoses (60). Athletes with elevated eating disorders symptoms identified during the initial screening (EDE-Q global score ≥1.68 for males and 2.30 for females) were invited to complete the EDE interview (version 17.0D; 61). The interview was administered by a single EDE trained and licensed clinician with a typical duration of 45 to 75 min.

#### Laboratory assessments

The laboratory procedures have been described in detail elsewhere (61) and are summarized in Figure 2. Briefly, body composition and bone mineral density (BMD) were assessed in a fasted and rested state using dual-energy x-ray absorptiometry (DXA) (Lunar iDXA, EnCore v18.10.105) at a local hospital beginning at approximately 07.00 h. All DXA measurements were performed in accordance with International Society for Clinical Densitometry recommendations (62). Low BMD was defined as a Z-score <−1 at the whole body, hip or lumbar spine, consistent with current REDs guidelines (2).

Participants subsequently arrived at the laboratory between 7.30–8.00 h. Upon arrival, a urine sample was collected to assess hydration status testing using urine-specific gravity refractometry (Digital Pocket Refractometer, ATAGO Co., Ltd., Tokyo, Japan). Stature was measured using a wall-mounted stadiometer (Seca Optima, Seca, Birmingham, UK), and body mass and body composition were assessed using a multifrequency bioelectrical impedance analyzer (InBody 770, Co, Ltd, Seoul, South Korea). Body mass index (BMI) was calculated as body mass (kg) divided by stature squared (m^2^).

RMR was subsequently measured by indirect calorimetry using an open circuit canopy hood system (Vyntus CPX open hood, CareFusion, Hochberg, Germany) following the protocol described by Melin, Tornberg (52). Participants rested quietly for 15 min before the assessment, followed by a 30 min measurement period. Data from the last 20 min were used for analysis to ensure steady state conditions. Measured RMR (mRMR) was calculated using the Weir equation (63), whereas predicted RMR (pRMR) was estimated using the Cunningham equation (64). Metabolic suppression was defined as an RMR ratio (mRMR/pRMR) <0.90 consistent with previous research in athlete populations (52, 65).

Following RMR assessments, blood pressure was measured using a digital handheld monitor (The Welch Allyn Connex® ProBP™ 3400, New York, USA). Venous blood samples were collected from an antecubital vein by a registered nurse. Blood samples were collected in BD Vacutainer serum tubes with clot activator and standard EDTA tubes (BD Biosciences, Franklin Lakes, NJ). Serum samples were allowed to clot for 60 min at room temperature before centrifugation (3,000 × g, 15 min at 4°C) whereas EDTA tubes were centrifuged immediately after blood sampling using the same setting. Serum and EDTA plasma were aliquoted into cryotubes (Sarstedt, Darmstadt, Germany), snap-frozen in liquid nitrogen, and stored at −80°C until analysis.

Serum concentrations of thyroid-stimulating hormone (TSH), free triiodothyronine (free T3), free thyroxine (free T4), estradiol, LH, prolactin and progesterone (females)/testosterone (males), insulin, cortisol, sex hormone-binding globulin (SHBG), and ferritin were measured using a commercially available chemiluminescent immunoassay (CLIA) on the Siemens Advia Centaur XPT platform (Siemens Healthineers, Erlangen, Germany), according to the manufacturer's instructions. Serum LDL cholesterol concentrations were measured using a commercially available elimination/catalase method from Siemens Advia Chemistry XPT (Siemens Healthineers, Erlangen, Germany), whereas total cholesterol concentrations were determined using a commercially available enzymatic method, according to the manufacturer's instructions. Serum IGF-1 concentrations were measured using a commercially available CLIA on the Siemens Immulite 2,000 XPi platform (Siemens Healthineers, Erlangen, Germany), according to the manufacturer's instructions.

To minimize analytic variability all samples for a given analyte were analyzed within a single analytic batch using the same assay-specific reagent, calibrator, and quality control lots, where applicable. Assay performance was monitored using manufacturer provided quality control materials at both low and high concentration levels. Analytical runs were accepted only when calibration and quality-control results met the laboratory's predefined acceptance criteria. Assay-specific limits of detection, analytic sensitivity, intra- and inter-assay coefficients of variation are provided in Supplementary Table S7. Laboratory reference intervals were used to determine the lowest quartiles of reference ranges for free T3 and free T4. In male athletes, free testosterone concentrations were calculated from total testosterone and SHBG concentrations using the Vermeulen equation, assuming an albumin concentration of 4.3 g/dL (66). Age-specific male reference ranges for total and calculated free testosterone were subsequently used to identify subclinical testosterone concentrations within the REDs classification framework (Table 1).

Prior to VO_2peak_ test, participants consumed a standardized breakfast based on body mass: 550 kcal (70 g carbohydrates, 25 g protein, 20 g fat) for athletes weighing <70 kg, and 800 kcal (100 g carbohydrate, 35 g protein, 30 g fat) for athletes weighing ≥70 kg. VO_2_peak was assessed on an electronically braked cycle ergometer (Monark LC7TT, Monark Exercise AB, Vansbro, Sweden) using a standardized ramp protocol described previously (61).

#### Classification of participants

Athletes were retrospectively classified as controls or REDs cases using a study specific severity/risk framework informed by the IOC REDs CAT2 and based on the presence and number of severe primary, primary, and secondary indicators (2) (Table 1). Athletes were categorized as controls if they had none/very low REDs risk, defined as the absence of primary indicators and no more than one secondary indicator. Athletes meeting criteria for mild, moderate-to-high, or very high/extreme REDs severity/risk were classified as REDs cases (28) (Table 2). Within the classification framework, each severe primary indicator was weighted as equivalent to two primary indicators. Mild REDs severity/risk was defined as the presence of one or two primary indicators and no more than one secondary indicator or the presence of up to two secondary indicators alone. Moderate-to-high severity/risk was defined as the equivalent of three primary indicators with no more than one secondary indicator, or two primary indicators combined with at least two secondary indicators. Very high/extreme severity/risk was defined as the equivalent of four or more primary indicators, or three primary indicators in combination with at least two secondary indicators. Potential indicators were summarized descriptively but were not scored or included in the IOC REDs CAT2-informed severity/risk stratification.

**Table 2:** Risk assessment of REDs based on the prevalence of clinical indicators distribution of potential REDs indicators across IOC REDs CAT2 severity/risk categories.

Risk classification	Criteria	*n* (%)	No. of potential REDs indicators, *n* (%)
Controls (none/very low risk)	0 primary and ≤1 secondary indicator	20 (46.5)	0: 3 (15.0)
			1: 7 (35.0)
			2: 5 (25.0)
			3: 4 (20.0)
			5: 1 (5.0)
Mild	1 primary+≤2 secondary, or 2 primary+≤1 secondary	18 (41.9)	0: 1 (5.6)
			1: 6 (33.3)
			2: 5 (27.8)
			3: 1 (5.6)
			4: 2 (11.1)
			5: 2 (11.1)
			6: 1 (5.6)
Moderate to high	Equivalent of 3 primary points ^a^	2 (4.7)	2: 1 (66.7)
			4: 1 (33.3)
Very high/extreme	Equivalent of ≥4 primary points ^b^	3 (9.3)	2: 3 (100)
All REDs cases	Meeting CAT2 mild, moderate to high, or very high/extreme severity	23 (53.5)	—

A severe primary indicator was counted as equivalent to two primary indicators. Percentages in the *n* (%) column are based on the 43 included athletes, whereas percentages in the potential indicator column, *n* (%) are based on participants within each severity/risk category. Potential indicators were not scored or included in IOC REDs CAT2 severity/risk stratification.

a Observed equivalent of 3 primary points: 1 severe primary +1 primary with or without 1 secondary indicator, or 3 primary +1 secondary indicators.

b Observed equivalent of ≥4 primary points: 1 severe primary +2 primary with or without 1 secondary indicator. CAT2, Clinical Assessment Tool-Version 2; EDE-Q, Eating Disorder Examination Questionnaire; LEAF-Q, Low Energy Availability in Females Questionnaire; NR, not reported; REDs, Relative Energy Deficiency in Sport.

Several participants specific circumstances required predefined modifications to the REDs classification process. One participant presented with a clinically verified eating disorder unrelated to dietary restraint, together with exercise dependence, but did not meet criteria for any primary, secondary, or other potential REDs indicators. Consequently, this participant was classified as a control. One female athlete classified with mild REDs severity/risk based on eating disorder and secondary amenorrhea, was subsequently found to have a testosterone-to-SHBG ratio above the clinical reference range, consistent with PCOS, and was therefore excluded from the study. Another female athlete classified with mild REDs severity/risk, based on low free T3 concentrations together with three potential indicators (low insulin, high cortisol, and gastrointestinal symptoms), was found to be in the first week of pregnancy at the time of testing, and was also excluded. Three participants had TSH concentrations above the clinical reference range, with free T3 and free T4 concentrations within the clinical reference range, consistent with subclinical hypothyroidism (67). These athletes were retained in the analyses, however thyroid hormones were not considered in the REDs classification process (control, *n* = 1; mild REDs, *n* = 1; very high/extreme REDs, *n* = 1). In addition, one participant classified with mild REDs severity/risk had a diagnosis of familial hypercholesterolemia. Consequently, total and LDL cholesterol values were excluded from the REDs classification for this participant.

#### Assessment of dietary intake and energy expenditure

Following the laboratory assessments, participants completed a 7-day free living assessment of energy expenditure in their home environment. Dietary intake was concurrently assessed over four days, including three weekdays and one weekend day, using weighed dietary records.

##### Dietary intake

Participants were instructed to weigh and register all foods, beverages, and other energy-containing items consumed and to document the time of each eating occasion. An electronic kitchen scale (OBH Nordica) was provided to facilitate accurate dietary recording. Trained research staff subsequently entered all dietary records into the validated nutrient-analysis software DietistNet (Kost och Näringsdata AB, Bromma, Sweden) to quantify daily energy intake and macronutrient consumption.

The plausibility of self-reported energy intake was evaluated using the Goldberg cut-off method described by Black (68). Individual energy intake-to-RMR ratios were compared with the lower 95% confidence limit derived from the duration of dietary recording, measured RMR, and an assumed physical activity level (PAL) representative of elite athletes. Participants with energy intake-to-RMR ratios below this threshold were classified as potential low energy reporters, rather than confirmed under-reporters, as the Goldberg method has limited sensitivity at the individual level and cannot distinguish dietary misreporting from true low energy intake. Four athletes were identified as potential low energy reporters, including two athletes classified with REDs and two control athletes. Both controls presented with one potential REDs indicator (hypotension or gastrointestinal symptoms). Because potential indicators were not included in the IOC REDs CAT2 severity/risk classification and because the Goldberg cut-off was applied solely as a dietary report plausibility check, all four athletes were retained in the analyses.

##### Energy expenditure

Participants logged the timing of all structured training sessions over seven consecutive days to capture total weekly training load and wore a heart rate monitor during each session. Individual linear heart rate–energy expenditure relationships, derived from V˙O_2peak_ testing, were used to estimate EEE for each session (69). Excess post-exercise oxygen consumption (EPOC) was modelled as 5% and 3% of net EEE during the first and second post-exercise hours, respectively (33, 34).

Hourly RMR was calculated from pRMR (unadapted) rather than mRMR (adapted) to reduce the risk of underestimating energy requirements. Sleeping energy expenditure was taken as 90% of pRMR during reported sleep hours.

Non-exercise activity thermogenesis (NEAT) was assessed via a tri-axial accelerometer (Actigraph wGT3X-BT) worn from waking to bedtime on the same days as dietary logging and removed for showering, swimming, and training sessions.

Diet-induced thermogenesis (DIT) was modelled as 10% of energy intake and allocated to hourly postprandial periods using the Reed and Hill (70) formula:

$$\mathrm{DIT}(T)\;=\;175.9\;\times \;T\;\times \;e^{-T/1.3}$$

### Calculations of within-day energy balance

WDEB was calculated on an hourly basis across the monitoring period. For each hour, WDEB was defined as:

$$\mathrm{WDE}B_{\mathrm{hour}}=\mathrm{EI}\;\text{-}\;(\mathrm{pRMR}\;\mathrm{or}\;\mathrm{Sleep}\;\mathrm{EE}+\mathrm{DIT}+\;\mathrm{EEE}+\mathrm{EPOC}+\mathrm{NEAT})$$

where pRMR was applied during waking hours and sleeping energy expenditure during sleep, yielding the unadapted WDEB. To characterize the WDEB profile for each athlete, summary WDEB variables were derived according to established approaches (22, 33–35). We first quantified total exposure to each energetic state by calculating the total number of hours per day spent in negative WDEB (WDEB <0 kcal), within the sex-specific balance range (WDEB_[±300/400]_), and positive WDEB (WDEB >0 kcal).

To account for the buffering capacity of liver glycogen, sex-specific thresholds (±WDEB_300/400_) were applied, defined as an energy deficit <−300 kcal or surplus > +300 kcal for females and <−400 kcal or > +400 kcal for males. These cut-offs correspond to the predicted amount of usable liver glycogen in female and male athletes (21, 22) and provide a more conservative characterization of estimated energetic deficit or surplus; thus, an athlete was not classified as below or above the sex-specific threshold until the hourly negative or positive WDEB exceeded these values. Using these thresholds, we calculated the total number of hours per day spent below the deficit cut-off (−WDEB_300/400_) and above the surplus cut-off (+WDEB_300/400_), as well as hours spent in EB (WDEB_[±300/400]_), defined as WDEB between −300 and +300 kcal for females and −400 and +400 kcal for males.

Because sustained energetic deficit may be more physiologically relevant than brief fluctuations, we also derived indices of consecutive exposure to within-day energy deficit, defining consecutive hours as blocks of at least three continuous hours. We computed the total number of consecutive hours in negative WDEB (−WDEB_*consec_) and, using the sex-specific thresholds, consecutive hours below the deficit cut-off (−WDEB_300/400∗consec_). In addition, we quantified the magnitude of the most extreme hourly deviations by determining, for each day, the largest single-hour energy deficit and surplus (kcal·h^−1^) and then averaging these daily maxima across the recording period. For comparison with traditional approaches, total daily EB and daily EA were also derived. All WDEB-related variables were calculated for each day and averaged across valid recording days for analysis.

### Statistical analysis

Statistical analyses were performed using jamovi version 2.7.15 and RStudio version 2026.04.0 (Posit Software, PBC, Boston, MA, USA). Continuous variables were screened for outliers and assessed for normality using histograms, Q–Q plots, and the Shapiro–Wilk test. Data are presented as mean ± SD for normally distributed variables and as median (25th–75th percentile) for non-normally distributed variables. Categorical variables are presented as *n* (%) or n/N (%) where denominators differed by sex or data availability.

Comparisons between athletes classified with REDs and controls were performed using independent-samples t-tests for normally distributed continuous variables and Mann–Whitney *U*-tests for non-normally distributed continuous variables. Welch's correction was applied where appropriate when homogeneity of variance was not met. Categorical variables were compared using chi-square tests or Fisher's exact tests, as appropriate. Effect sizes were reported as Cohen's d for normally distributed continuous variables, rank-biserial correlation (rᵦ) for non-normally distributed continuous variables, the phi coefficient (*φ*) for 2 × 2 categorical variables, and Cramér's V for categorical variables with more than two categories. Cohen's d was interpreted as small, medium, and large using thresholds of 0.20, 0.50, and 0.80, respectively (71, 72). Rank-biserial correlations were interpreted as very small (<0.10), small (0.10–0.29), moderate (0.30–0.49), and large (≥0.50) based on absolute values (73).

Associations between predefined WDEB metrics, 24 h energy variables, and REDs-relevant indicators were assessed using Spearman's rank correlation coefficients (*ρ*), with two-sided *p*-values. Correlation coefficients were interpreted as small, medium, and large using thresholds of 0.10, 0.30, and 0.50, respectively (71, 72). Correlation analyses used pairwise complete observations, and no imputation was performed. Benjamini–Hochberg false discovery rate (BH-FDR) adjustment was applied as a sensitivity analysis for exploratory correlation analyses. Correlations with *p* < 0.05 before adjustment were considered nominally significant, whereas *q* < 0.05 was used for FDR-adjusted inference. Approximate 95% confidence intervals for nominally significant Spearman correlations were calculated using Fisher's z transformation.

Sex-specific analyses were restricted to REDs-relevant indicators with sex-specific biological relevance, scoring, or thresholds, according to the IOC REDs CAT2 framework (2, 28). Female-only indicators included menstrual-function variables, anovulation, and LEAF-Q-derived gastrointestinal symptoms. Male-only indicators included total testosterone, clinical/subclinical low testosterone, and LEAM-Q-derived low libido. EDE-Q global score was analyzed in all participants as a continuous variable, whereas elevated EDE-Q scores were classified using sex-specific cut-offs (≥2.30 for female and ≥1.68 for male athletes). In correlation analyses, gastrointestinal scores were analyzed in female athletes only, and total testosterone was analyzed in male athletes only. Statistical significance was set at *p* < 0.05.

## Results

### Participant characteristics and REDs classification

Participant demographics, selected REDs-related clinical indicators, and sport/training characteristics are summarized in Table 3, and a summary of the retrospective classification of REDs including frequency of potential indicators is presented in Table 2.

**Table 3:** Participants demographic details and REDs indicators.

Measure	All (*n* = 43)	REDs (*n* = 23)	Controls (*n* = 20)	*P* value	Effect size (d/rb/*φ*)
Sex, *n* (%)				0.068	0.295
Male	17 (39.5)	6 (26.1)	11 (55.0)		
Female	26 (60.5)	17 (73.9)	9 (45.0)		
Demographics					
Age (yrs)	24 (22–28)	24 (22–28)	24 (21–28)	0.714	0.067
Height (cm)	174 ± 9	170 ± 8	178 ± 8	0.004	0.932
Weight (kg)	70 ± 13	65 ± 10	76 ± 13	0.004	0.967
BMI (kg/m^2^)	23 ± 3	22 ± 2	24 ± 3	0.043	0.658
Body Fat (%)	15 (11–18)	15 (13–18)	13 (10–18)	0.592	0.098
Fat Mass (kg)	9 (8–12)	9 (8–12)	10 (9–12)	0.408	−0.150
FFM (kg)	55 (50–69)	53 (49–59)	63 (53–76)	0.024	−0.404
VO_2_peak (mL·kg^−1^·min^−1^)	50 ± 7	50 ± 8	49 ± 7	0.691	−0.121
Primary indicators					
Free T3 (pmol/L)	5.3 ± 0.9	4.8 ± 0.8	5.7 ± 0.6	<0.001	1.221
Free T4 (pmol/L)	15.1 (13.7–16.2)	13.9 (12.6–15.2)	16.0 (15.3–16.6)	<0.001	−0.609
Testosterone, males (nmol/L), *n* = 17	24.1 ± 7.6	22.5 ± 9.3	24.9 ± 6.9	0.593	0.309
Males with low testosterone, n/N (%)	1/17 (5.9)	1/6 (16.7)	0/11 (0.0)	0.353	0.339
Whole-body BMD Z-score	2.1 ± 1.1	2.0 ± 1.4	2.2 ± 0.8	0.469	0.216
Total hip BMD Z-score	1.1 ± 1.1	1.0 ± 1.2	1.4 ± 0.8	0.203	0.385
Lumbar spine L1–L4 BMD Z-score	0.8 ± 1.3	0.5 ± 1.4	1.2 ± 1.2	0.080	0.542
EDE-Q global score	1.23 (0.48–1.87)	1.86 (1.19–3.54)	0.58 (0.28–1.09)	<0.001	0.676
Secondary indicators					
Total cholesterol (mmol/L)	4.5 (3.8–5.3)	4.7 (4.0–5.8)	4.0 (3.5–4.7)	0.020	0.417
LDL cholesterol (mmol/L)	2.8 (2.2–3.1)	2.9 (2.4–3.3)	2.5 (1.7–2.9)	0.047	0.357
MDI score	18.1 ± 8.3	20.2 ± 9.1	15.7 ± 6.7	0.066	−0.565
Depression symptoms, n/N (%)	6/43 (14.0)	6/23 (26.1)	0/20 (0.0)	0.023	0.376
Sport and training characteristics					
Sport category, *n* (%)				0.144	0.437
Team ball games	14 (32.6)	5 (21.7)	9 (45.0)		
Weight category	6 (14.0)	4 (17.4)	2 (10.0)		
Technical	3 (7.0)	2 (8.7)	1 (5.0)		
Aesthetic	5 (11.6)	1 (4.3)	4 (20.0)		
Strength	4 (9.3)	2 (8.7)	2 (10.0)		
Endurance	11 (25.6)	9 (39.1)	2 (10.0)		
Weight-sensitive sport, *n* (%)				0.131	0.252
Yes	22 (51.2)	14 (60.9)	8 (40.0)		
No	21 (48.8)	9 (39.1)	12 (60.0)		
Training volume (h/week)	12 (10–16)	14 (11–16)	12 (10–16)	0.392	0.154

BMI, body mass index; BMD, bone mineral density; EDE-Q, Eating Disorder Examination Questionnaire; FFM, fat-free mass; LDL, low-density lipoprotein; MDI, Major Depression Inventory; REDs, Relative Energy Deficiency in Sport; T3, triiodothyronine; T4, thyroxine. d, Cohen's d; rb, rank-biserial correlation; φ, phi coefficient. Data are presented as mean ± SD for normally distributed variables and as median (25th–75th percentile) for non-normally distributed variables. *p* < 0.05 indicates statistical significance.

The initial online screening identified 19 athletes (44%) as being at risk of REDs. Among female athletes 17 met at least one screening criterion, including a LEAF-Q score ≥8 (*n* = 16) and/or an EDE-Q global score ≥2.30 (*n* = 9). Among male athletes, two exceeded the male specific EDE-Q global score cutoff (≥1.68). All 11 participants with sex-specific elevated EDE-Q global score, completed the EDE interview. Based on the clinical interview, one athlete demonstrated disordered eating behavior, one met the diagnosis of a non-restrictive eating disorder subtype, seven were diagnosed with atypical anorexia nervosa, and two with atypical bulimia nervosa.

Application of the retrospective IOC REDs CAT2-informed classification framework (Tables 1, 2) identified 20 participants (47%) as controls with none or very low risk of REDs, of whom 45% were female. Three of these athletes had been identified as at risk during the initial online screening.

Twenty-three participants (54%) were classified with REDs, of whom 74% were female. Notably, seven athletes classified with REDs, including five males, had not been identified as at risk during the initial online screening.

Among female participants, total LEAF-Q scores were significantly higher in those classified with REDs than in controls [10.0 (8.0–15.0) vs. 5.0 (4.0–6.0), *p* = 0.027]. Using the LEAF-Q threshold of ≥8, 14 of 17 females classified with REDs (82%) and 2 of 9 female athlete controls (22%) exceeded the at-risk cutoff.

Among athletes classified with REDs, most were categorized as having mild severity/risk (*n* = 18, 78%). Two athletes were classified with moderate to high severity, and three with very high to extreme severity/risk. The most common primary and secondary REDs indicators were low free T3 or free T4 concentration (*n* = 13), elevated sex specific EDE-Q global score (*n* = 10), menstrual disturbances (*n* = 9), symptoms of moderate to severe depression (*n* = 6), low BMD (*n* = 7), elevated total and/or LDL cholesterol concentrations (*n* = 5), history of stress fracture (*n* = 3), and low testosterone concentration (*n* = 1).

Among the nine female participants with menstrual disturbances, four reported prolonged secondary amenorrhea, three secondary amenorrhea, and two oligomenorrhea. All had prolactin, progesterone, LH/follicle-stimulating hormone ratio, testosterone, and testosterone/SHBG ratio values within clinical reference ranges. Among the eight eumenorrheic female athletes classified with REDs, five demonstrated evidence of anovulatory cycles.

The distribution of sex and sport categories is presented in Table 3. Athletes classified with REDs included a higher proportion of female athletes than controls [17/23 (73.9%) vs. 9/20 (45.0%)]. Endurance athletes were also more prevalent in the REDs group [9/23 (39.1%) vs. 2/20 (10.0%)], and participation in weight-sensitive sport was descriptively more common among athletes classified with REDs.

Compared with controls, participants classified with REDs were shorter and had lower body mass, BMI and FFM, consistent with the greater proportion of female athletes in this group. No significant differences were observed for age, body fat, training volume or VO_2_peak.

Potential REDs indicators among controls and participants classified with REDs are summarized in Table 4. Compared with controls, participants classified with REDs displayed a lower RMR ratio, higher cortisol concentrations, and a higher cortisol-to-insulin ratio. No other potential REDs indicator differed significantly between the groups.

**Table 4:** Potential REDs indicators among participants diagnosed with REDs and controls.

Measure	All (*n* = 43)	REDs (*n* = 23)	Controls (*n* = 20)	*P* value	Effect size (d/rb/φ)
Behavioral and reproductive indicators					
EAI score, points	21.2 ± 3.5	22.1 ± 2.9	20.1 ± 3.8	0.056	−0.616
Exercise dependence, *n* (%)	10 (23.3)	6 (26.1)	4 (20.0)	0.728	0.072
Eumenorrheic females with anovulatory cycles, n/N (%)	7/18 (38.9)	4/9 (44.4)	3/9 (33.3)	1.000	0.114
Cardiovascular, metabolic and endocrine indicators					
Resting HR, beats/min	48 ± 8	47 ± 6	49 ± 9	0.367	0.285
Bradycardia, *n* (%)	3 (7.0)	2 (8.7)	1 (5.0)	1.000	0.072
Systolic BP, mm Hg	112 ± 10	112 ± 11	113 ± 9	0.696	0.119
Diastolic BP, mm Hg	65 (63–69)	66 (63–70)	65 (63–68)	0.874	0.030
Hypotension, *n* (%)	4 (9.3)	2 (8.7)	2 (10.0)	1.000	0.022
RMRratio	0.90 ± 0.16	0.85 ± 0.15	0.96 ± 0.16	0.028	0.698
mRMR (kcal⋅kg^−1^ FFM⋅d^−1^)	30 ± 5	29 ± 5	31 ± 5	0.097	0.521
Low RMR, *n* (%)	22 (51.2)	14 (60.9)	8 (40.0)	0.227	0.208
Metabolic and endocrine indicators					
GI-score (females)	2.5 (2.0–4.8)	3.0 (2.0–5.0)	2.0 (2.0–4.0)	0.763	0.078
Females with GI score ≥2, n/N (%)	21/26 (80.8)	13/17 (76.5)	8/9 (88.9)	0.628	0.150
Insulin (mU/L)	4.6 (3.7–6.0)	3.9 (3.3–5.7)	4.9 (4.2–6.2)	0.082	−0.313
Low insulin (mU/L), *n* (%)	6 (14.0)	5 (21.7)	1 (5.0)	0.192	0.241
IGF-1 (µg/L)	171.0 (144.0–196.0)	165.0 (135.0–196.0)	172.5 (156.2–198.5)	0.458	−0.135
Low IGF-1 (µg/L), *n* (%)	9 (20.9)	6 (26.1)	3 (15.0)	0.467	0.136
Cortisol (nmol/L)	530.4 ± 153.8	596.0 ± 146.3	455.1 ± 127.9	0.002	−1.021
Cortisol:insulin ratio (nmol/L per mU/L)	106.4 (77.5–167.8)	162.9 (92.0–215.8)	87.0 (64.4–107.5)	0.002	0.548
High cortisol (nmol/L), *n* (%)	2 (4.7)	2 (8.7)	0 (0.0)	0.491	0.206
Ferritin (µg/L)	26.0 (14.7–42.5)	26.0 (13.6–41.2)	25.6 (17.1–46.5)	0.827	−0.041
Low ferritin (µg/L), *n* (%)	6 (14.0)	2 (8.7)	4 (20.0)	0.393	0.163

REDs, Relative Energy Deficiency in Sport; BP, blood pressure; EAI, Exercise Addiction Inventory; FFM, fat-free mass; GI, gastrointestinal; HR, heart rate; IGF-1, insulin-like growth factor-1; LEAF-Q, Low Energy Availability in Females Questionnaire; mRMR, measured RMR; RMR, resting metabolic rate; RMR ratio, measured-to-predicted RMR ratio; d, Cohen's d; rᵦ, rank-biserial correlation; φ, phi coefficient. Data are presented as mean ± SD or median (25th–75th percentile), unless otherwise indicated. Categorical variables are presented as *n* (%) or n/N (%) when denominators are subgroup specific. *p* < 0.05 indicates statistical significance.

The distribution of potential REDs indicators within each group is presented in Table 2. Among controls, two presented with a single secondary indicator (depression symptoms or exercise dependence), whereas 50% displayed two or more potential indicators. Three eumenorrheic controls demonstrated anovulatory cycles. Of these, two had also two additional potential indicators (gastrointestinal problems, low RMR or hypotension) and one displayed four additional potential indicators (gastrointestinal problems, low RMR, exercise dependence and clinically low ferritin).

Among participants with mild REDs severity, 61% presented with two or more potential indicators (Table 2). All participants with moderate-to-high and very high/extreme REDs severity/risk presented with two or more potential indicators.

### Dietary intake and within-day energy balance metrics

Dietary characteristics, EB, EA, and WDEB metrics are presented in Table 5. Ten participants were excluded from dietary, EB, EA, and WDEB analyses because of incomplete accelerometer, nutritional, and/or training data. Consequently, the available-case analysis comprised 17 athletes classified with REDs (70.6% female) and 16 controls (43.8% female).

**Table 5:** Dietary intake and energy balance metrics among controls and REDs participants.

Variable	All (n = 33)	REDs (*n* = 17)	Controls (*n* = 16)	*P-*value	Effect size (d/rb)
24 h EB and EA					
24 h EB (kcal)^a^	−388 (−720–−154)	−534 (−720–−291)	−209 (−709–350)	0.087	−0.353
24 h EA (kcal/kg FFM)^b^	33 (28–41)	32 (28–36)	40 (30–49)	0.038	−0.426
Dietary intake					
24 h EI (kcal)	2,858 ± 850	2,492 ± 538	3,246 ± 959	0.011	0.979
Carbohydrates (g/day)	302 (259–382)	266 (225–364)	339 (288–388)	0.094	−0.346
Carbohydrates (g/kg/day)	4.7 ± 1.6	4.4 ± 1.3	4.9 ± 1.9	0.443	0.275
Carbohydrates (E%)	44 (40–51)	45 (42–52)	43 (38–49)	0.439	0.162
Fiber (g/day)	27 (22–39)	31 (24–46)	25 (20–30)	0.177	0.279
Fiber (g/1,000 kcal)	2.6 (1.7–3.3)	2.7 (2.6–4.4)	1.8 (1.5–2.5)	0.004	0.596
Protein (g/day)	119 ± 34	108 ± 32	131 ± 34	0.056	0.695
Protein (g/kg/day)	1.7 ± 0.4	1.7 ± 0.5	1.8 ± 0.4	0.610	0.178
Protein (E%)	17 ± 4	17 ± 4	17 ± 3	0.521	−0.225
Fat (g/day)	108 (83–132)	86 (75–109)	124 (107–140)	0.004	−0.588
Fat (g/kg/day)	1.6 ± 0.4	1.4 ± 0.3	1.8 ± 0.5	0.027	0.829
Fat (E%)	35 (29–39)	34 (28–38)	38 (32–41)	0.126	−0.316
Energy expenditure					
24 h EE (kcal)^a^	3,151 (2,771–3,818)	3,069 (2,912–3,229)	3,578 (2,733–4,099)	0.305	−0.213
24 h EE (kcal)^b^	2,999 (2,649–3,889)	2,952 (2,744–3,025)	3,410 (2,635–4,143)	0.155	−0.294
Exercise EE (kcal/day)	794 (445–1,086)	726 (478–972)	873 (436–1,141)	0.871	−0.037
DIT (kcal/day)	276 ± 86	241 ± 56	314 ± 97	0.014	0.935
EPOC (kcal/day)	64 (36–87)	58 (38–78)	70 (35–90)	0.871	−0.037
NEAT (kcal/day)	303 (212–410)	279 (207–346)	388 (246–539)	0.081	−0.360
RMR (kcal/day)	1,662 ± 442	1,491 ± 319	1,843 ± 490	0.023	0.856
Within-day EB variables					
Hourly EB	−586 ± 1,349	−1,045 ± 1,078	−98 ± 1,466	0.045	0.740
WDEB <0 kcal (h/day)	19.0 (7.0–22.5)	21.5 (16.5–22.5)	8.6 (3.8–20.9)	0.028	0.452
WDEB >0 kcal (h/day)	5.0 (1.5–17.0)	2.5 (1.5–7.5)	15.4 (3.1–20.2)	0.028	−0.452
−WDEB_consec_ (h/day)	19.0 (6.8–22.5)	21.5 (15.8–22.5)	8.2 (3.8–20.9)	0.024	0.463
−WDEB_300/400_ (h/day)	15.0 (4.0–20.2)	18.2 (13.5–20.8)	6.8 (1.8–19.3)	0.061	0.386
−WDEB_300/400*consec_ (h/day)	14.5 (3.5–20.0)	17.8 (12.5–20.2)	6.2 (1.3–18.5)	0.058	0.390
WDEB _[± 300/400]_ (h/day)	2.8 (1.7–4.8)	3.4 (1.6–6.0)	2.5 (1.9–4.2)	0.589	0.114
+WDEB_300/400_ (h/day)	3.5 (0.0–13.5)	0.8 (0.0–3.5)	11.5 (0.9–18.0)	0.017	−0.485
Largest hourly deficit (kcal) (kcal/day)	−1,297 (−2,279–−640)	−1,468 (−2,047–−1,178)	−708 (−2,428–184)	0.135	−0.309
Largest hourly surplus (kcal)	206 ± 1,406	−364 ± 988	811 ± 1,554	0.016	0.909

DIT, diet-induced thermogenesis; EA, energy availability; EB, energy balance; EE, energy expenditure; EI, energy intake; EPOC, excess post-exercise oxygen consumption; Exercise EE, exercise energy expenditure; FFM, fat-free mass; NEAT, non-exercise activity thermogenesis; RMR, resting metabolic rate; WDEB, within-day energy balance.

a Calculated using predicted/unadapted RMR (pRMR).

b Calculated using measured/adapted RMR (mRMR).

Data are presented as mean ± SD for normally distributed variables and as median (25th–75th percentile) for non-normally distributed variables. *p* < 0.05 indicates statistical significance.

Athletes classified with REDs, demonstrated lower 24 h EA and lower reported 24 h energy intake than controls. In contrast, no significant between group differences were observed for 24 h EB, EE, EEE, EPOC, and NEAT. Dietary composition differed between groups. Participants classified with REDs reported lower total and relative fat intake, and had higher fiber density than controls, whereas carbohydrate and protein intake did not differ significantly between groups.

**Figure 3:**
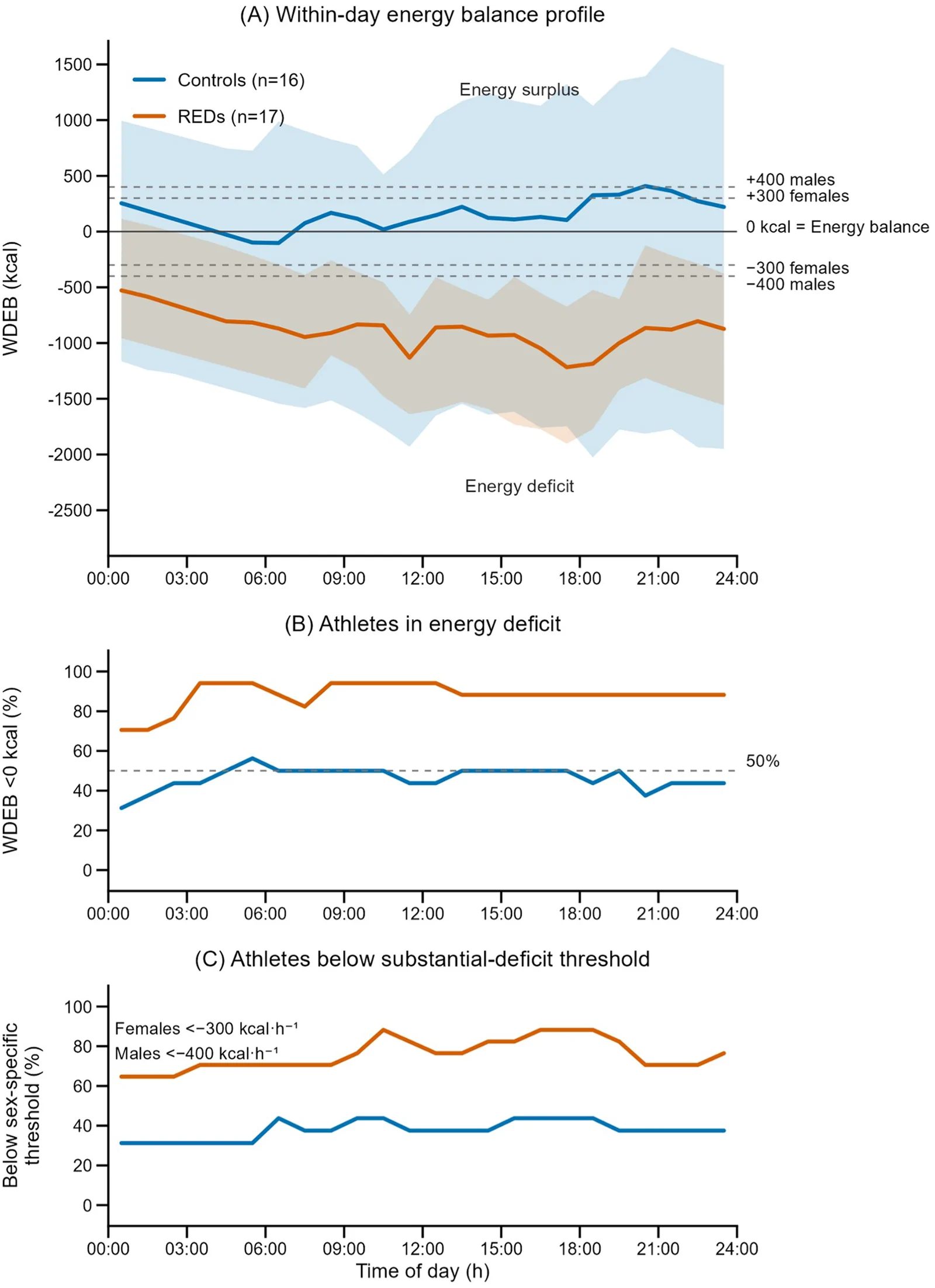
Within-day energy balance profiles across 24 h by REDs status. **(A)** Group-level within-day energy balance (WDEB) profiles in athletes classified with REDs (*n* = 17) and controls (*n* = 16). Lines represent group medians and shaded areas represent the interquartile range. The solid horizontal line indicates energy balance (0 kcal); values above 0 indicate energy surplus and values below 0 indicate energy deficit. Dashed lines indicate the sex-specific surplus and deficit thresholds. **(B)** Percentage of athletes with WDEB <0 kcal at each hour. **(C)** Percentage of athletes below the sex-specific deficit threshold at each hour. WDEB, within-day energy balance; REDs, Relative Energy Deficiency in Sport.

Analysis of WDEB revealed that athletes classified with REDs accumulated more hours in negative WDEB (WDEB <0 kcal) and fewer hours in positive WDEB (WDEB > 0 kcal) than controls (Table 5, Figure 3). They also experienced longer consecutive periods in negative WDEB (−WDEB_*consec_). Time spent below the sex-specific substantial deficit threshold (−WDEB_300/400_) and consecutive time below this threshold (−WDEB_300/400*consec_) did not differ between groups, whereas time spent above the sex-specific substantial surplus threshold (+WDEB_300/400_) and the largest hourly surplus were lower among participants classified with REDs.

### Associations between REDs indicators and within-day energy balance metrics

Correlation analyses for potential indicators are presented in Table 6. In the full WDEB sample, insulin was associated with 24 h EB and 24 h EA before BH-FDR adjustment; however, neither association remained significant after adjustment within the potential-indicator correlation family (both *q* > 0.05; Supplementary Tables S8, S9).

**Table 6:** Correlations between REDs potential indicators and within-day energy metrics. .

Variable	WDEB <0 kcal (h)		−WDEB_consec_ (h)		−WDEB_300/400_ (h)		–WDEB_300/400∗consec_ (h)		Largest hourly deficit (kcal)		24 h EB (kcal)		24 h EA (kcal/kg/FFM)	
	*ρ*	*P* value	ρ	*P* value	ρ	*P* value	ρ	*P* value	ρ	*P* value	ρ	*P* value	ρ	*P* value
Anthropometric/hemodynamic														
BMI	0.128	0.478	0.142	0.430	0.222	0.215	0.219	0.222	−0.333	0.058	−0.152	0.399	−0.179	0.319
Systolic BP	0.008	0.965	0.001	0.997	0.058	0.749	0.059	0.746	−0.203	0.258	0.020	0.912	−0.044	0.809
Diastolic BP	−0.096	0.596	−0.109	0.544	−0.161	0.372	−0.166	0.355	0.100	0.580	0.080	0.658	0.018	0.920
Resting HR	−0.061	0.736	−0.076	0.676	−0.066	0.716	−0.082	0.649	0.027	0.883	−0.054	0.764	−0.105	0.561
Metabolic/hematological potential indicators														
Ferritin	0.097	0.592	0.097	0.592	0.058	0.747	0.056	0.759	−0.036	0.842	0.061	0.737	0.083	0.645
Glucose	0.078	0.666	0.079	0.662	0.121	0.501	0.139	0.440	−0.221	0.216	−0.072	0.690	−0.095	0.598
Cortisol	0.280	0.114	0.279	0.116	0.235	0.188	0.225	0.208	−0.114	0.526	−0.003	0.987	−0.028	0.877
Cortisol:insulin ratio	0.158	0.381	0.165	0.358	0.143	0.429	0.123	0.494	−0.142	0.430	−0.240	0.179	−0.210	0.240
IGF-1	0.191	0.288	0.198	0.270	0.271	0.127	0.282	0.112	−0.194	0.279	−0.147	0.415	−0.138	0.444
Insulin	−0.034	0.850	−0.047	0.796	−0.057	0.755	−0.041	0.820	0.154	0.392	0.401	0.021*	0.347	0.048*
RMRratio	0.006	0.972	0.009	0.960	0.049	0.786	0.024	0.893	−0.103	0.567	0.121	0.504	0.130	0.471
RMR kcal/kg FFM	0.051	0.777	0.043	0.813	0.103	0.567	0.069	0.702	−0.131	0.466	0.025	0.890	0.046	0.799
Behavioral/symptom variables														
EAI score	0.102	0.572	0.122	0.497	0.045	0.806	0.063	0.728	−0.106	0.558	−0.178	0.322	−0.225	0.207
GI symptoms^a^ score^a^ (females)	0.027	0.914	0.028	0.909	0.010	0.967	0.042	0.865	−0.115	0.638	−0.039	0.874	0.027	0.914

WDEB <0 kcal (h/day), total number of hours per day spent in negative WDEB (EB <0 kcal·h^−1^). −WDEB_consec_, total number of consecutive hours (blocks ≥ 3 h) spent in negative WDEB (EB <0 kcal·h^−1^). −WDEB_300/400_ (h/day), total number of hours per day below the sex-specific substantial deficit threshold (females: EB <−300 kcal·h^−1^; males: EB <−400 kcal·h^−1^), reflecting depletion beyond estimated liver glycogen buffering capacity. −WDEB_300/400*consec_, total number of consecutive hours (blocks ≥ 3 h) spent below the sex-specific substantial deficit threshold (females: EB <−300 kcal·h^−1^; males: EB <−400 kcal·h^−1^). Largest hourly deficit, most negative single hourly EB value observed during the day (kcal). 24 h EB, 24 h energy balance. 24 h EA (kcal·kg FFM^−1^·d^−1^), 24 hour energy availability (kcal·kg FFM^−1^·d^−1^). BMI, body mass index; BP, blood pressure; HR, heart rate; RMR; resting metabolic rate; RMR ratio, measured/predicted RMR (mRMR/pRMR); IGF-1, insulin-like growth factor 1; GI, gastrointestinal symptoms.

a GI symptom correlations were computed in females only (*n* = 19). *p* < 0.05 indicates statistical significance.

In exploratory sensitivity analyses among female athletes with WDEB data (*n* = 19), EAI score was inversely associated with 24 h EB (*ρ* = −0.496, *p* = 0.031), BMI was inversely associated with 24 h EA (*ρ* = −0.460, *p* = 0.048), and cortisol-to-insulin ratio was positively associated with time spent in negative WDEB (*ρ* = 0.489, *p* = 0.034) and consecutive time in negative WDEB (*ρ* = 0.512, *p* = 0.025). None of these associations remained significant after BH-FDR adjustment (all q > 0.05; Supplementary Tables S8, S9).

## Discussion

This cross-sectional study examined selected endocrine and metabolic markers of energy deficiency in relation to WDEB in elite Swedish national-team athletes from multiple sport disciplines. In this cohort, more than half of the athletes were retrospectively classified with REDs using an IOC REDs CAT2-informed framework, and most athletes classified with REDs were female. Although most REDs cases were classified as mild, the majority exhibited multiple potential REDs indicators. Notably, half of the controls also presented with two or more potential REDs indicators, suggesting that REDs-related signs and symptoms may exist along a continuum and are not fully captured by categorical risk classifications.

Compared with controls, athletes classified with REDs displayed higher cortisol concentrations, and a higher cortisol-to-insulin ratio. They reported lower energy intake, had less energy-dense dietary characteristics, similar 24 h EB while lower estimated 24 h EA, and more pronounced within-day energy deficits than controls. WDEB analyses further revealed longer and more frequent periods of negative WDEB, and reduced exposure to surplus patterns above the sex-specific thresholds. Together these findings suggest that athletes classified with REDs experienced a more catabolic hormone profile and greater exposure to within-day energy deficits despite similar estimates of total daily EB.

In exploratory correlation analyses, insulin, the cortisol-to-insulin ratio, BMI, and exercise dependence symptoms were associated with selected 24 h EB, 24 h EA or WDEB metrics. However, none of these associations remained significant after BH-FDR adjustment.

Collectively, these findings suggest that REDs classification in this cohort was characterized by a multidimensional and individually variable constellation of physiological, behavioral, and psychological features. The results further indicate that WDEB may provide complementary context beyond dietary intake, 24 h EA and 24 h EB. Finally, the heterogenous endocrine and metabolic findings reinforce the notion that that biological responses to energy deficiency are biomarker specific and may require sex-specific interpretation within the clinical evaluation of REDs.

### Classification of relative energy deficiency in sport

In this cohort of elite athletes, 53% of athletes were retrospectively classified with REDs; however, the relatively small sample size and potential inclusion bias should be considered when interpreting this prevalence estimates. Nevertheless, the observed prevalence is comparable to the 45% reported in a cohort of Canadian elite athletes from mixed sport disciplines (*n* = 213, 67% female) (37) assessed using the IOC REDs CAT2 framework (28). Direct comparison between cohorts should be made with caution because almost half of the female athletes in the Canadian study reported hormonal contraception use and menstrual cycle status was not included in the scoring framework, whereas menstrual and ovulatory function formed part of the assessment in the present study. Despite these methodological differences, the high prevalence of observed in both cohorts may reflect the greater representation of female athletes, consistent with the sex-specific distribution seen here and in the Canadian cohort, where 35% and 41% of male athletes, respectively, were classified with REDs.

In both studies, most athletes classified with REDs were categorized within the mild severity/risk category. Importantly, most mild cases in the present study also exhibited multiple additional potential indicators, providing further clinical context and supporting the notion of an underlying physiological, behavioral or psychological burden. However, 39% of athletes classified with mild REDs had no or not more than one potential indicator, highlighting the heterogeneity of REDs presentations and the challenges associated with categorizing athletes using the discrete risk thresholds. Conversely, half of the athletes classified as controls (none/very low risk) exhibited two or more potential indicators that, under the original REDs CAT (74) or contemporary Female Athlete Triad frameworks (75), might have contributed to a higher risk classification. This overlap was particularly evident among the three eumenorrheic control athletes who demonstrated anovulatory cycles in combination with additional potential indicators.

Because potential indicators were summarized descriptively rather than incorporated into the IOC REDs CAT2-informed severity/risk stratification, these findings suggest that categorical classification may not fully capture borderline, subclinical or evolving REDs presentations. This observation is consistent with the current conceptualization of REDs as a spectrum of physiological and behavioral adaptations that vary according to the severity, duration and frequency, as well as individual modifiers such as biological sex, reproductive status, training load, dietary composition, psychological stress and susceptibility to energy deficiency (2, 7, 23, 76). Taken together, these findings reinforce the importance of interpreting REDs within a comprehensive, multi-marker clinical framework rather than relying solely on categorical risk classification (2, 23, 28). They also highlight the need for future studies to evaluate the prognostic value of currently unscored indicators and to determine whether additional physiological, behavioral, or reproductive markers should be incorporated into future iterations of the REDs CAT framework.

### Interpretation of dietary, energy availability, and energy balance findings

The lower estimated EA observed in athletes classified with REDs in the present study was primarily attributed to lower reported total energy intake, rather than differences in estimated total or exercise-related EE. These findings are consistent with the current understanding that intentional or unintentional under-fueling is a key contributor to LEA and the development of REDs (2, 77). In addition, athletes classified with REDs reported lower fat intake and a higher fiber density, a dietary pattern that resembles previous observations in female athletes with functional hypothalamic amenorrhea (5, 6). Such dietary habits may reduce the overall energy density of the diet, particularly when characterized by a high intake of fiber rich foods and a low intake of fat and carbohydrate-rich foods, thereby making it more difficult to meet the substantial energy demands of high-performance training (78).

However, these dietary findings should be interpreted with caution. Athletes classified with REDs included a higher proportion of female athletes and were descriptively more represented in endurance and weight-sensitive sports than controls. Consequently, some of the observed differences in dietary intake may reflect sex- and sport-specific dietary practices rather than REDs classification alone. This possibility is supported by evidence indicating a higher prevalence of restricted eating, disordered eating behavior and eating disorders among female compared with male elite athletes (79). Moreover, sociocultural and sport specific body-ideals may influence dietary behaviors differently in female than male athletes, with greater emphasis on thinness and leanness in female and muscularity in male athletes (80). These pressures may contribute to distinct patterns of dietary restraint, food choice, and body-weight regulation that influence energy intake independently of REDs status.

### WDEB as a complementary lens on energy deficiency

The WDEB analyses in the present study provided complementary insights beyond traditional 24 h EA and EB. Athletes classified with REDs accumulated more time in negative WDEB, experienced longer consecutive periods of energy deficits, and exhibited directionally greater exposure to sex-specific deficit thresholds despite the absence of a significant between-group difference in 24 h EB. These findings are consistent with previous WDEB studies demonstrating that athletes with similar 24 h EA or EB values may nevertheless exhibit markedly different within-day energy distribution pattern (33, 34). The observation of lower estimated 24 h EA in the REDs group, despite comparable 24 h EB, likely reflects the fact that EA and EB capture distinct aspects of energy status. Whereas EA quantifies the energy remaining after EEE relative to FFM, EB reflects the difference between energy intake and total daily energy expenditure. In mixed-sex cohorts, variation in body size, FFM, sex distribution, and individual energy expenditure components may therefore contribute to differences in EA without necessarily producing corresponding differences in absolute 24 h EB. By incorporating the temporal distribution of energy intake and EE, WDEB extends these traditional metrics provides information regarding the timing, duration, and magnitude of estimated deficits and surpluses throughout the day.

Although athletes classified with REDs demonstrated endocrine and metabolic differences, insulin was the only biomarker that showed nominal association with 24 h EB and EA in the full WDEB sample. This finding may be biologically plausible given the sensitivity of insulin to recent energy intake, carbohydrate availability and substrate utilization (81, 82). However, the association did not remain significant following the BH-FDR adjustment and should therefore be regarded as exploratory. Similarly, none of the endocrine or metabolic potential indicators remained significantly associated with WDEB metrics after correction for multiple testing. These findings contrast with earlier studies reporting relationships between WDEB derived metrics and endocrine or metabolic adaptations to energy deficiency. In female endurance athletes (33) and male and female swimmers (35) prolonged within-day deficits have been associated with suppressed thyroid hormones and RMR. In male endurance athletes, WDEB metrics have been linked to suppressed RMR, higher cortisol and a lower testosterone-to-cortisol ratio (34). Several factors may explain why comparable associations were not observed in the present study. First, this cohort included both female and male athletes representing a wide range of sport disciplines, introducing substantial physiological behavioral heterogeneity. Second, the relatively small sample size limited statistical precision and power to detect potential meaningful associations. Thirdly, nearly half the athletes represented national teams in non-leanness sports including technical, strength and team sports, which may be characterized by different fueling practices, and body composition than endurance athletes, and further contribute to variability in physiological responses. Consequently, the lack of significant associations between WDEB and endocrine biomarkers should not be interpreted as evidence against their existence. Instead, it may reflect the combined influences of sample heterogeneity, limited statistical power, and the fact that EA, EB and WDEB estimates capture only a brief snapshot of athletes' habitual fueling practices and training demands. This interpretation is supported by recent findings in female soccer players, in whom commonly used LEA-risk indicators including low RMR ratio, low BMD, total LEAF-Q score ≥8, and symptoms of eating disorders (83)] were not consistently associated with measured EA or WDEB (36).

Taken together, these findings suggest that WDEB may provide clinically relevant contextual information beyond traditional 24 h EA and EB assessments by capturing the temporal distribution of energy intake and expenditure throughout the day. Nevertheless, the lack of robust endocrine associations in the present study indicates that the physiological relevance of specific WDEB patterns requires further investigation. Although longitudinal studies are needed to clarify these relationships, repeated measurement of true dietary intake and energy expenditure is methodologically complex, resource intensive, and places a substantial burden on athletes, making prolonged monitoring difficult to implement in both research and high-performance environments. Future work should therefore focus on identifying practical and valid surrogate markers that can complement or reduce reliance on intensive dietary and energy-expenditure assessments in REDs screening and monitoring.

### Endocrine, metabolic, and sex-specific interpretation

Athletes classified with REDs exhibited lower RMR ratio, higher cortisol concentrations, and a higher cortisol-to-insulin ratio than controls. These findings are consistent with aspects of the energy conserving and stress-related adaptations previously reported in athletes exposed to LEA, including female athletes (17, 33), and male athletes (84–86). Overall, 22 athletes (51%), including 19 females, exhibited a suppressed RMR ratio, of whom 14 (64%) were classified with REDs. However, all but one control with low RMR ratio also presented with one or more potential indicators. These findings highlights the limited specificity of RMR-related measures when interpreted in isolation, and supports concerns regarding the utility of the RMR ratio as a REDs as a stand-alone marker of REDs (37). Nevertheless, a suppressed RMR ratio remains an indicator of severe energy deficiency within the updated Female Athlete Triad framework (20), suggesting that its value may be greatest when interpreted alongside other clinical, physiological, and behavioral indicators.

Similarly, elevated cortisol concentrations and higher cortisol-to-insulin ratio may be compatible with stress-related and energy-conservation responses to LEA. However, cortisol is influenced by numerous factors beyond energy status, including recent training load, psychological stress, sleep quality, illness, injury, sampling conditions, and acute nutritional status (19, 34, 77). Consequently, cortisol should be interpreted within a broader clinical context and in conjunction with complementary biomarkers rather than as an isolated indicator of REDs. In the present study, the two athletes with clinically elevated cortisol concentrations were classified as having mild REDs severity/risk with multiple additional potential indicators and moderate to severe REDs severity/risk, respectively, further supporting the need for multi-marker interpretation. The cortisol-to-insulin ratio may provide additional context by integrating a stress-related catabolic marker with a biomarker reflecting recent nutritional status and substrate availability. Conceptually, this ratio may therefore offer greater insight into the balance between catabolic and anabolic processes than either marker alone. However, evidence remains limited, and clinically meaningful thresholds have yet to be established. Further research is therefore required before the cortisol-to-insulin ratio can be considered a valid indicator within REDs assessment frameworks.

Taken together, these findings should not be interpreted as evidence of a generalized endocrine disturbance among athletes classified with REDs. Rather, only selected endocrine and metabolic markers differed between groups, reinforcing the concept that physiological responses to energy deficiency are biomarker specific and highly variable between individuals. Accordingly, endocrine and metabolic findings should be interpreted within the broader context of dietary intake, energy status, training load, reproductive function, and overall clinical assessment rather than as independent diagnostic indicators of REDs.

### Clinical and practical implications

The overlap of REDs-related indicators across severity/risk categories, encompassing behavioral, psychological, reproductive, metabolic, and endocrine domains, reinforces the need for comprehensive, multi-indicator assessment strategies in elite sport. The present findings support current recommendations that REDs should be evaluated through an integrated clinical framework rather than through isolated biomarkers or screening tools (Ackerman et al., (23); Mountjoy et al., (2); Stellingwerff et al., (28)). Within such a framework, dietary assessment and WDEB analyses may provide valuable contextual information that complements clinical, physiological, and endocrine evaluation since it may help identify potentially modifiable fueling behaviors that are not captured by daily EA or EB estimates alone (21, 33–35). Dietary analyses provide complementary insight into food choices, dietary composition, and eating patterns, including low-energy-density dietary behaviors that may contribute to chronic under-fueling despite apparently adequate food volume (Melin et al., (5)). Together, these approaches may help guide individualized nutrition interventions aimed at improving energy distribution across the day rather than focusing solely on total daily energy intake.

Nevertheless, the practical implementation of EA, EB, and WDEB assessment remains challenging. Accurate estimation requires detailed dietary records, precise measurement or modeling of energy expenditure, and substantial participant compliance, all of which impose considerable logistical and psychological burdens on athletes and support staff. Moreover, repeated longitudinal assessment of true dietary intake and energy expenditure is subject to well-recognized methodological limitations, including reporting error, behavioral reactivity, training-period variability, and uncertainties in energy-expenditure estimation. As a result, intensive long-term monitoring may be difficult to justify in research, as well as in clinical, and high-performance sport settings.

Accordingly, WDEB and dietary metrics should currently be considered complementary contextual tools rather than stand-alone diagnostic measures of REDs. In the present study, WDEB findings should not be interpreted as evidence that WDEB independently identifies REDs status. Rather, the observed associations may have been influenced by differences in sex distribution, sport type, dietary-reporting accuracy, training context, and the assumptions underlying modeled energy-expenditure calculations. Future research should therefore focus on determining whether WDEB-derived metrics provide incremental clinical value beyond existing REDs assessment frameworks and on developing valid, low-burden monitoring approaches that are feasible for routine use in elite athletes.

### Strengths and limitations

Key strengths of this study include the inclusion of Swedish national-team athletes from multiple sports and both sexes, the integration of WDEB metrics with endocrine and metabolic assessments, and the application of the IOC REDs CAT2-informed classification framework. In addition, clinically relevant REDs indicators were evaluated using established assessment protocols, including ovulatory function monitoring in eumenorrheic athletes and clinician verified eating-disorder diagnoses, in line with current REDs consensus recommendations (2, 28, 37).

Several limitations should also be considered. The cross-sectional design precludes causal inference regarding the relationships among REDs classification, endocrine and metabolic biomarkers, and WDEB metrics. Although the study initially was powered to detect clinically relevant differences in sex hormone concentrations based on previous work (11), female and male athletes were unevenly distributed across the REDs and control groups. Consequently, planned sample size targets were not achieved within all comparison subgroup limiting statistical power and precision for sex-stratified, sport specific, subgroup, and multivariable analyses. Therefore, nonsignificant findings, particularly those relating endocrine markers and subgroup analyses, should be interpreted as inconclusive rather than as evidence of physiologically similarity between groups.

The unequal distribution of sex and sport disciplines may also have influenced observed differences in anthropometry, reported dietary intake, RMR-related variables, EA, WDEB metrics, and selected endocrine/metabolic markers. Sex-specific indicators, including menstrual-function variables, anovulation, testosterone, libido-related variables, and ferritin where relevant, were interpreted with sex-specific frameworks. However, several biochemical potential indicators, including insulin, IGF-1, cortisol, glucose, and RMR ratio, were evaluated using common thresholds. Accordingly, pooled analysis should be interpreted with caution, while recognizing the potential sex dependent physiological responses to energy deficiency.

Although the prevalence of REDs was broadly consistent with findings from larger elite-athlete cohorts (37), and recent meta-analytic evidence (18), the multistage recruitment process from online screening to clinical assessment may have introduced selection bias. Athletes concerned about REDs-related symptoms may have been more likely to volunteer for clinical assessment, potentially affecting prevalence estimates and group composition.

Dietary intake, EA, EB and WDEB estimates should also be interpreted considering cumulative measurement uncertainty. Despite the use of weighed dietary records, under-reporting, missed items, and reactivity cannot be excluded. Furthermore, the relatively short assessment period may reflect recent dietary intake and training practices rather than the longer-term exposure likely to underlie many REDs-related adaptations (7, 19, 23). This limitation is particularly relevant because endocrine, reproductive, and skeletal consequences of energy deficiency often develop over weeks to months rather than days. In validation studies using doubly labelled water, self-reported dietary assessment in athletes has been reported to underestimate energy intake by approximately 19% on average corresponding to roughly 600 kcal/day (87). Although four athletes were identified as potential low energy reporters using the Goldberg method, these athletes were retained because the method cannot distinguish reflects true under fueling from reporting bias. Excluding them could therefore have removed clinically relevant low-intake cases, reduced statistical precision, and introduced additional selection bias.

WDEB estimation includes multiple methodological uncertainties because it combines measured, estimated, and modeled variables, including predicted RMR, diet-induced thermogenesis, excess post-exercise oxygen consumption, heart-rate-derived exercise energy expenditure, and accelerometer-derived non-exercise activity thermogenesis (7, 19, 23). Accurate quantification of EEE is particularly challenging in a mixed-sport elite-athlete cohort because energy-cost prediction varies according to exercise mode, intensity, movement efficiency, and training structure. Individualized heart rate-EE calibrations were used to improve EEE estimation accuracy, but they cannot eliminate errors associated to non-steady-state exercise, sport-specific movement patterns, or interindividual variability in physiological responses. More broadly, device-based estimates of daily energy expenditure may differ from criterion methods such as metabolic chambers or doubly labeled water by approximately 100–600 kcal per day (88). Moreover, prediction of RMR using the Cunningham equation carries an expected error of approximately 10% (64). Consequently, cumulative uncertainty across energy intake, EEE, NEAT, DIT, EPOC, and RMR estimates may influence both absolute WDEB values and observed associations with endocrine and metabolic outcomes. Predicted rather than measured RMR was used to estimate unadapted energy requirements and avoid incorporating metabolic suppression into energy-requirement calculations. However, this modeling choice may also have influenced the magnitude of estimated deficits.

A *post hoc* scenario-based sensitivity analysis suggested that the overall pattern of greater within-day energy deficits among athletes classified with REDs was robust under plausible variation in selected input assumptions (Supplementary Table S10). Nevertheless, this exploratory analysis did not formally quantify cumulative error propagation across all modeled components. Accordingly, WDEB estimates should be interpreted as indicators of temporal energy-status patterns rather than precise measures of hourly EB.

The correlation analyses, including the exploratory female-only sensitivity analyses, should likewise be interpreted cautiously because of the modest sample size, available-case analysis, and the large number of comparisons examined. After BH-FDR adjustment, no nominally significant correlation remained statistically significant following correction for multiple testing. These findings should therefore be considered hypothesis-generating rather than confirmatory. Future studies should include larger and more balanced cohorts enabling adequately powered sex- and sport-specific analysis, multivariable modeling approaches. Formal error-propagation analysis may also help quantify the influence of uncertainty in WDEB inputs and modelling assumptions on estimated within-day energy-status patterns.

Finally, endocrine and metabolic biomarkers were assessed from a single standardized fasted morning fasting sample. Although pre-assessment standardized reduced variability related to recent feeding and exercise, a single sampling point cannot fully capture longer-term or dynamic endocrine status. Consequently, these biomarkers should be interpreted as complementary components of a broader REDs assessment rather than definitive indicators of chronic endocrine adaptation.

## Conclusion

In this cohort of Swedish national-team athletes, more than half were retrospectively classified with REDs, with a predominance of female athletes. Compared with controls, athletes classified with REDs demonstrated lower estimated 24 h EA, lower reported energy intake, greater exposure to estimated negative WDEB patterns, and selected metabolic and endocrine differences that may be consistent with energy deficiency when interpreted within a broader clinical context. However, WDEB should not be interpreted as an independent indicator of REDs status. Importantly, the substantial overlap of potential REDs indicators across severity/risk categories, including athletes with mild REDs presenting few additional potential indicators and athletes classified with none/very low risk presenting multiple potential indicators, suggests that categorical classification systems may not fully capture the heterogenous, evolving, and continuum-like nature of REDs presentations. Collectively, these findings support the use of integrated, multi-indicator assessment approaches that combine clinical, behavioral, reproductive, endocrine, metabolic, dietary and energy-status information. Within such as framework, WDEB and dietary characterization may help contextualize the broader REDs profile and identify potentially modifiable fueling patterns. Future research should determine which combinations of primary, secondary, and potential indicators, together with contextual measures of energy status such as WDEB, provide the greatest clinical utility for REDs risk stratification and monitoring in larger, longitudinal, sex balanced and sport specific cohorts.

## Data Availability

The raw data supporting the conclusions of this article will be made available by the authors, without undue reservation.
