# Supplementary material for: Endocrine markers of energy deficiency and within-day energy balance metrics in elite athletes
Source: Front Sports Act Living. 2026 Aug 12;8:1894602. doi: 10.3389/fspor.2026.1894602 (PMC13506786; doi:10.3389/fspor.2026.1894602)
Supplement: Supplementary file 1 [file Datasheet1.docx]

Supplementary Material

**Supplementary Table 7.** Analytical characteristics of biochemical assays.

| **Analyte** | **Platform** | **Assay principle/method** | **Lower analytical limit/sensitivity** | **Intra-assay CV**  Low- and high-level concentration | **Inter-assay CV**  Low- and high-level concentration |
| --- | --- | --- | --- | --- | --- |
| Ferritin | ADVIA Centaur XPT | Chemiluminescent (C) immunoassay | 0.5 µg/L | 1.67% at 27.46; 2.49% at 327.1 µg/L | 0.31% at 28.91; 2.74% at 122.08 µg/L |
| Cholesterols |  |  |  |  |  |
| Total | ADVIA Chemistry XPT | Enzymatic method | 0.26 mmol/L | 0.55% at 2.73; 0.48% at 6.48 mmol/L | 0.45% at 2.79; 0.29% at 6.55 mmol/L |
| LDL | ADVIA Chemistry XPT | Elimination/  catalase method | 0.21 mmol/L | 0.31% at 1.77; 0.41% at 3.97 mmol/L | 0.59% at 1.76; 0.88% at 4.04 mmol/L |
| TSH | ADVIA Centaur XPT | C immunoassay | 0.008 mIU/L | 1.04% at 0.96; 1.34% at 8.14 mIU/L | 0.68% at 0.70; 0.47% at 4.85 mIU/L |
| Free T3 | ADVIA Centaur XPT | C immunoassay | 0.3 pmol/L | 1.11% at 3.29; 0.79% at 8.89 pmol/L | 1.64% at 3.47; 0.22% at 9.18 pmol/L |
| Free T4 | ADVIA Centaur XPT | C immunoassay | 1.3 pmol/L | 3.33% at 15.13; 3.38% at 30.68 pmol/L | 1.01% at 12.91; 1.93% at 30.81 pmol/L |
| Estradiol | ADVIA Centaur XPT | C immunoassay | 69.8 pmol/L | 3.10% at 173.5; 1.20% at 7259.0 pmol/L | 3.64% at 145.19; 0.30% at 3555.89 pmol/L |
| LH | ADVIA Centaur XPT | C immunoassay | 0.07 IU/L | 2.88% at 4.21; 2.67 at 20.0 IU/L | 0.23% at 20.74; 1.00 at 65.05 IU/L |
| Prolactin | ADVIA Centaur XPT | C immunoassay | 6.4 mIU/L | 1.69% at 6.99; 0.99% at 14.72 mIU/L | 0.60% at 6.90; 2.80% at 14.94 mIU/L |
| Testosterone | ADVIA Centaur XPT | C immunoassay | 0.24 nmol/L | 3.34% at 5.50; 4.58% at 49.93 nmol/L | 1.76% at 6.26; 0.91% at 39.81 nmol/L |
| SHBG | ADVIA Centaur XPT | C immunoassay | 1.8 nmol/L | 2.98% at 3.78; 1.97% at 19.67 nmol/L | 1.21% at 21.02; 0.65% at 45.54 nmol/L |
| Insulin | ADVIA Centaur XPT | C immunoassay | 0.5 mIU/L | 1.65% at 13.57; 1.82% at 199.42 mIU/L | 9.41% at 13.00; 8.83% at 194.01 mIU/L |
| IGF-1 | IMMULITE 2000 XPi | C immunoassay | 24.9 µg/L | 5.64% at 59.6; 2.27% at 180.3 µg/L | 2.21% at 67.80; 0.35% at 297.60 µg/L |
| Cortisol | ADVIA Centaur XPT | C immunoassay | 8.6 nmol/L | 3.43% at 46.7; 3.16% at 687.7 nmol/L | 3.08% at 142; 4.13% at 1140.01 nmol/L |

*Note.* Values are reported as lower analytical limits/sensitivities and coefficients of variation (CVs) at low and high concentration levels. Intra-assay CV refers to within-run precision, and inter-assay CV refers to between-run precision. CV, coefficient of variation; free T3, triiodothyronine; free T4, thyroxin; IGF-1, insulin-like growth factor 1; LDL, low-density lipoprotein; LH, luteinizing hormone; SHBG, sex hormone-binding globulin; TSH, thyroid-stimulating hormone.

**Supplementary Table 8.** Benjamini–Hochberg false discovery rate summary for exploratory correlation analyses.

| **Correlation family** | **No. tests** | **Nominal p<0.05** | **FDR q<0.05** | **Smallest q** | **Interpretation** |
| --- | --- | --- | --- | --- | --- |
| Full-sample potential indicators | 98 | 2 | 0 | 0.980 | No correlation remained significant after FDR |
| Female-only REDs indicators | 63 | 0 | 0 | 0.962 | No nominal or FDR-significant correlations |
| Female-only potential indicators | 98 | 4 | 0 | 0.664 | No correlation remained significant after FDR |

*Note.* Benjamini–Hochberg false discovery rate (BH-FDR) adjustment was applied within each predefined exploratory correlation family. Nominal p < 0.05 refers to unadjusted two-sided p-values, whereas FDR q < 0.05 refers to correlations that remained significant after BH-FDR adjustment. “No. tests” represents the total number of correlation tests evaluated within each family, and “smallest q” represents the lowest FDR-adjusted q-value observed within that family. Full-sample analyses were based on participants with within-day energy balance (WDEB) data (n = 33), whereas female-only analyses were based on females with WDEB data (n = 19). Analyses used available cases, and no imputation was performed. BH-FDR, Benjamini–Hochberg false discovery rate.

**Supplementary Table 9.** Exploratory correlation analyses with FDR-adjusted inference.

| **Family** | **Variable** | **Metric** | **ρ** | **Approx. 95% CI** | **P value** | **FDR q** |
| --- | --- | --- | --- | --- | --- | --- |
| Full-sample potential indicators | Insulin | 24-h EB (kcal) | 0.401 | 0.067 to 0.654 | 0.021 | 0.980 |
| Full-sample potential indicators | Insulin | 24-h EA (kcal/kg FFM) | 0.347 | 0.004 to 0.617 | 0.048 | 0.980 |
| Female-only potential indicators | BMI | 24-h EA (kcal/kg FFM) | -0.460 | -0.756 to -0.007 | 0.048 | 0.664 |
| Female-only potential indicators | EAI score | 24-h EB (kcal) | -0.496 | -0.775 to -0.054 | 0.031 | 0.664 |
| Female-only potential indicators | Cortisol:insulin ratio | WDEB <0 kcal (h) | 0.489 | 0.045 to 0.772 | 0.034 | 0.664 |
| Female-only potential indicators | Cortisol:insulin ratio | −WDEB_consec_ (h) | 0.512 | 0.075 to 0.784 | 0.025 | 0.664 |

*Note.* This table presents exploratory correlations with nominal unadjusted p < 0.05. Values are Spearman’s rank correlation coefficients (ρ), approximate 95% confidence intervals, unadjusted two-sided p-values, and Benjamini–Hochberg false discovery rate (BH-FDR)-adjusted q-values. Full-sample analyses were based on participants with within-day energy balance data (n = 33), whereas female-only analyses were based on females with within-day energy balance data (n = 19). BH-FDR, Benjamini–Hochberg false discovery rate; BMI, body mass index; CI, confidence interval; EA, energy availability; EAI, exercise addiction inventory; EB, energy balance; FFM, fat free mass; WDEB, within-day energy balance.

**Supplementary Table 10.** Post hoc scenario-based sensitivity analysis of within-day energy balance metrics.

| **Metric** | **Baseline REDs vs controls** | **Combined conservative REDs vs controls** |
| --- | --- | --- |
| 24-h EA, kcal/kg FFM | 32.4 vs 40.1 | 44.9 vs 52.0 |
| WDEB <0 kcal, h | 21.5 vs 8.6 | 19.0 vs 6.5 |
| −WDEB_consec_, h | 21.5 vs 8.3 | 18.8 vs 5.9 |
| −WDEB_300/400*consec_, h | 17.8 vs 6.3 | 13.0 vs 4.5 |
| Largest hourly deficit, kcal | −1468 vs −708 | −1093 vs −452 |
| 24-h EB, kcal | −534 vs −209 | 113 vs 842 |

*Note.* Values are medians for athletes classified with REDs versus controls. The baseline scenario used the original within-day energy balance (WDEB) calculations. Sensitivity scenarios included increasing hourly energy intake by 19% with proportional scaling of diet thermogenesis (DIT), reducing predicted resting metabolic rate (RMR)/sleep energy expenditure by 10%, reducing activity-derived exercise energy expenditure (EEE), excess post-exercise oxygen consumption (EPOC) and non-exercise activity thermogenesis (NEAT) by 10%, and a combined conservative scenario including all three adjustments. The sensitivity analysis was exploratory and did not constitute formal error-propagation modelling. EA, energy availability; EB, energy balance; FFM, fat-free mass.
